# Complete Genome Sequences of One Salt-Tolerant and Petroleum Hydrocarbon-Emulsifying *Terribacillus saccharophilus* Strain ZY-1

**DOI:** 10.3389/fmicb.2022.932269

**Published:** 2022-07-28

**Authors:** Zhaoying Su, Shicheng Yang, Mingchang Li, Yu Chen, Shaojing Wang, Yuan Yun, Guoqiang Li, Ting Ma

**Affiliations:** Key Laboratory of Molecular Microbiology and Technology, Ministry of Education, College of Life Sciences, Nankai University, Tianjin, China

**Keywords:** *Terribacillus saccharophilus*, strain ZY-1, complete genome, comparative genomics, salt-tolerant

## Abstract

Salt tolerance is one of the most important problems in the field of environmental governance and restoration. Among the various sources of factors, except temperature, salinity is a key factor that interrupts bacterial growth significantly. In this regard, constant efforts are made for the development of salt-tolerant strains, but few strains with salt tolerance, such as *Terribacillus saccharophilus*, were found, and there are still few relevant reports about their salt tolerance from complete genomic analysis. Furthermore, with the development of the economy, environmental pollution caused by oil exploitation has attracted much attention, so it is crucial to find the bacteria from *T. saccharophilus* which could degrade petroleum hydrocarbon even under high-salt conditions. Herein, one *T. saccharophilus* strain named ZY-1 with salt tolerance was isolated by increasing the salinity on LB medium step by step with reservoir water as the bacterial source. Its complete genome was sequenced, which was the first report of the complete genome for *T. saccharophilus* species with petroleum hydrocarbon degradation and emulsifying properties. In addition, its genome sequences were compared with the other five strains that are from the same genus level. The results indicated that there really exist some differences among them. In addition, some characteristics were studied. The salt-tolerant strain ZY-1 developed in this study and its emulsification and degradation performance of petroleum hydrocarbons were studied, which is expected to widely broaden the research scope of petroleum hydrocarbon-degrading bacteria in the oil field environment even in the extreme environment. The experiments verified that ZY-1 could significantly grow not only in the salt field but also in the oil field environment. It also demonstrated that the developed salt-tolerant strain can be applied in the petroleum hydrocarbon pollution field for bioremediation. In addition, we expect that the identified variants which occurred specifically in the high-salt strain will enhance the molecular biological understanding and be broadly applied to the biological engineering field.

## Introduction

The aerobic, spore-forming, Gram-positive, rod-shaped, moderately halophilic bacteria are a taxonomically diverse group whose members have been isolated from various environments (An et al., 2007). Previous studies have shown that halotolerant and moderately halophilic bacteria consist of several phylogenetically distinct lineages scattered throughout the genus of *Bacillus* based on phylogenetic and chemotaxonomic analyses (Ash et al., 1991). Based on the 16S rRNA gene sequence and chemotaxonomic analysis, several phylogenetically distinct lineages in the genus of *Bacillus* have been revealed (Ash et al., 1991). Subsequently, many spore-forming bacteria have been placed in separate genera and some moderately halophilic spore-forming bacteria were described as the genus of *Halobacillus* (Spring et al., 1996).

The genus of *Terribacillus* at present includes at least three species. Previous studies have shown that *Terribacillus saccharophilus* and *Terribacillus halophilus* were isolated from field soil in Japan (An et al., 2007), and *Terribacillus goriensis* DSM 18252T was isolated from coastal water on the east coast of Korea (Kim et al., 2007; Krishnamurthi and Chakrabarti, 2008). Afterward, Gregor Grass et al. isolated eight species from heroin samples, which were then proved to be the endospore-forming bacteria of *T. saccharophilus* (Kalinowski et al., 2018). Studies have also shown that the main phenotypic characteristics of *Terribacillus* genus are the formation of ellipsoidal endospores, gelatin liquefaction, the absence of H_2_S, indole production, and nitrate reduction (Liu et al., 2010). The GC content is generally between 43.0 and 46.0%, and the major fatty acids are anteiso-C_15:0_ and anteiso-C_17:0_ (An et al., 2007; Krishnamurthi and Chakrabarti, 2008). In total, three moderately halophilic bacterial strains designated YI7-61T, IA7, and DB2 were isolated from sediments [12.1–15.4% (w/v) NaCl] of Aiding Salt Lake in Xinjiang, China (Liu et al., 2005). Moderately halophilic bacteria contain a heterogeneous physiological group of microorganisms consisting of different genera and species that can grow in saline environments (Ventosa et al., 1998). The spore-forming moderately halophilic bacteria are an important group and were formerly placed in the genus of *Bacillus*, which were mostly found in the marine or hypersaline environments (Claus and Berkeley, 1986). Previous studies have paid more attention to the salt tolerance of *Terribacillus*, but few studies have shown that they can also play a role in the field of petroleum hydrocarbon degradation or emulsification.

Thus, in this study, a *T. saccharophilus* strain, named ZY-1, was isolated from the produced water of Changqing oil field (China). The biological features of ZY-1 were explored, and the complete genome was also sequenced. Furthermore, the genome sequences of other five strains from the same genus including *T. saccharophilus*.7521 (7521), *T. saccharophilus*.7518 (7518), *T. saccharophilus*.7503-2 (7503-2), *T. saccharophilus*.7517-W (7517-W), and *T. saccharophilus*.7528 (7528) were compared with ZY-1, respectively, which would provide a promising resource to research the salt tolerance mechanism even the petroleum hydrocarbon pollution remediation of *T. saccharophilus*.

## Materials and Methods

### Characteristics of ZY-1

Strain ZY-1 of *T. saccharophilus* isolated from reservoir water was identified using previously reported methods (Su et al., 2022). LB liquid medium (tryptone 10 g/L, yeast extract 5 g/L, and NaCl 10 g/L) was used to culture ZY-1, and LB solid medium was prepared by adding an extra 1.5% ager powder into LB liquid medium to harvest monoclonal bacteria. The optimum growth temperature (including 25, 30, 37, 42, and 45°C) and optimum pH (including pH 5.5, pH 6.0, pH 6.5, pH 7.0, pH 7.2, pH 7.5, pH 8.0, pH 8.5, pH 9.0, pH 9.5, pH 10.0, pH 10.5, and pH 11.0) of ZY-1 were all explored (OD_600_) in LB medium by using a spectrophotometer and a multifunctional microplate reader (Enspire; United States), respectively, and the utilization of carbon sources were tested by using the basic medium [K_2_HPO_4_ 4.8 g/L, KH_2_PO_4_ 1.5 g/L, (NH_4_)_2_SO_4_ 1.0 g/L, MgSO_4_⋅7H_2_0 0.2 g/L, yeast extract 0.01 g/L, PH 7.2, extra add 1% carbon source]. The API-50CH system is usually used to identify the utilization of different carbohydrates (Rath et al., 1995), and the API-ZYM (Analytab Products Inc., Plainview, NY, United States) system is a semiquantitative micro-method, which allows rapid determination of 19 enzymatic reactions (Slots, 1981). Thus, API-50 CH and API-ZYM microtest galleries were separately used to determine the physiological and biochemical characteristics, including the utilization of different carbohydrates and the activity of exogenous enzymes.

Biofilms are often considered as a source of a problem in the medical fields and in the industry even in the environments (Banat et al., 2014). Therefore, the formation of biofilms in LB medium at different salinities were studied on polystyrene plates by using crystal violet staining based on these methods (Pratt and Kolter, 2010). Since ZY-1 was isolated from the reservoir water, which belongs to an extreme environment, the emulsifying and petroleum hydrocarbon degradation properties were all studied. The crude surfactant was first produced with sucrose as the carbon source, and the products were then obtained by using previous methods (Zhang et al., 2015; Caruso et al., 2019; Hu et al., 2020). EI_24_ was finally measured to evaluate the emulsifying properties (Cooper and Goldenberg, 1987). To explore the degradation performance of ZY-1 on crude oil, 2% crude oil was added to the basic medium as the sole carbon source. The content of total n-alkanes (C13–C36) in petroleum hydrocarbons after culturing for 7 days at 6% NaCl was detected by using crude oil as the sole carbon source. The methods for crude oil degradation were the same as described in previous studies (Su et al., 2022). The detection method was referenced as described (Li et al., 2021), and the same experiments without adding bacteria were used as controls. In addition, to more intuitively characterize the morphological structure of ZY-1 at different salinities, the scanning electron microscope (SEM, QUANTA 200, Czech) was used to observe the morphological structure of bacteria without salt and with an additional 10% NaCl (Shehadat et al., 2018).

### Genomic DNA Isolation, Sequencing, Assembly, and Annotation

The genomic DNA was extracted using a TIANamp Bacteria DNA Kit (TIANGEN Biotech Co., Ltd, Beijing, China) following the manufacturer’s instructions, and the quality and quantity of purified genomic DNA were determined by using a NanoDrop 2000 spectrophotometer (Thermo Scientific, MA, United States).

The genome of *T. saccharophilus* strain ZY-1 was sequenced using the PacBio sequel II and DNBSEQ platform at Beijing Genomics Institute (BGI, Shenzhen, China). In total, four SMRT cells with *zero-mode waveguide* arrays of sequencing were used by the PacBio platform to generate the subread set. The PacBio subreads (length < 1 kb) were removed, and the program Canu v1.5^1^ was used for self-correction. Draft genomic unitigs, which are uncontested groups of fragments, were assembled using the canu, a high-quality corrected circular consensus sequence subread set. To improve the accuracy of the genome sequences, GATK v1.6-13^2^ was used to make single-base corrections. Glimmer 3.02 software (Delcher, 1999; Delcher et al., 2007) was used for the genetic prediction of assembly results. rRNA library was used to find rRNA, or RNAmmer v1.2 software (Karin et al., 2007) was used to predict rRNA. In addition, the tRNA region and tRNA secondary structure were all predicted by tRNAscan v1.3.1 software (Lowe and Eddy, 1997). SRNA was obtained by comparing Infernal software with the Rfam 9.1 database (Gardner et al., 2008)^3^, and tandem repetition sequences are predicted by Tandem Repeat Finder v4.04 software (Gary, 1999). According to the length of repetition units, the number of microsatellites and small satellite sequences was screened.

Gene annotation was mainly based on amino acid sequence alignment. The best hit was abstracted using the blast alignment tool for functional annotation. In total, seven databases including Kyoto Encyclopedia of Genes and Genomes v89.1, Clusters of Orthologous Groups (COG) v2020-11-25, Non-Redundant Protein Database databases, Swiss-Prot [18], Gene Ontology (GO), TrEMBL, and EggNOG were used for general function annotation. It is worth noting that the information on COG annotation was shown as an example to better understand the gene function in this article.

### Genome Comparison

TYPE (strain) Genome Server^4^ was used to identify the species of ZY-1, and the whole sequenced genomes of the other five *T. saccharophilus* strains were selected and downloaded (2021/12/15) from the NCBI database for comparative genomic analysis. The positions on the genetic maps of both species are similar, which could be used to analyze the changes in genetic gene loci caused by recombination, transposition, and other mechanisms between the sequencing genome and the reference genome. Synteny refers to the interlocking genes in a species genome, and it is also linked to the genome of another species. The synteny usually includes the nucleotide level and amino acid level. Compared with the amino acid level, synteny analysis of the nucleotide level could show the insertion and deletion of sequences. This analysis can be used to obtain the structural variation (chromosome rearrangement, etc.) of genomes between strains during the evolutionary process, such as the position change of gene clusters with similar functions in different strains. Therefore, the synteny including *T. saccharophilus*.ZY-1 and *T. saccharophilus*.7521, *T. saccharophilus*.ZY-1 and *T. saccharophilus*.7518, *T. saccharophilus*.ZY-1 and *T. saccharophilus*.7503-2, *T. saccharophilus*.ZY-1 and *T. saccharophilus*.7517-W, *T. saccharophilus*.ZY-1, and *T. saccharophilus*.7528 were respectively performed using MUMmer v3.22^5^ and BLAST Core/Pan genes and clustered by the CD-HIT v4.6.6 rapid clustering of similar proteins software, with a threshold of 50% pairwise identity and 0.7 length difference cutoff at the nucleotide level and amino acid level, respectively.

Gene family was constructed and integrated by multiple software: align the protein sequence in BLAST, eliminate the redundancy by solar, and carry out gene family clustering treatment for the alignment results with Hcluster_sg software. In addition, the common genes and specific genes of these six strains were also analyzed and compared. Genes that share are usually called the common genes (mostly genes that are necessary for the growth of the strain), and genes that are unique to the organisms are often called unique genes. The protein gene sets of all strains were analyzed by CD-HIT v4.6.6 (Limin et al., 2012) cluster analysis, and the final gene set of the cluster was regarded as a pan-gene set. The sequences of each sample in the cluster were extracted as a core gene set, and the specific gene set in the product is the specific base set. The pan-gene set removes the core gene set, and the specific gene set is the dispensable gene set. The commonly used methods of phylogenetic tree construction mainly include the distance-based algorithm (UPGMA and NJ algorithm), maximum minimization algorithm, and maximum likelihood value method. The software used includes PAUP, Mega, TreeBeST, and PHYLIP. In this study, the phylogenetic tree was constructed by TreeBeST using the method of neighbor joining (NJ) method.

### Direct Link to Deposited Data and Information to Users

The BioProject and BioSample designations for *T. saccharophilus* strain ZY-1 are PRJNA828369 and SAMN27652359, respectively. The raw genome sequences have been deposited in GenBank under the accession number CP096209 on 26 April 2022. Strain ZY-1 of *T. saccharophilus* is available from the Key Laboratory of Molecular Microbiology and Technology, College of Life Sciences, Nankai University.

### Statistical Analysis

Each experiment was performed with three replicates. The values represent the means ± SD. Statistical analysis was performed using software Origin version 9.0.

## Results and Discussion

### Characteristics of ZY-1

**FIGURE 1:**
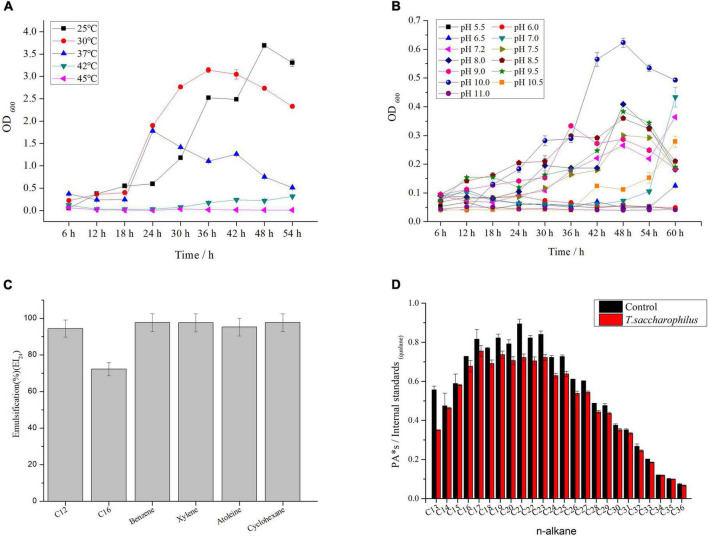
**(A)** Growth curve of ZY-1 at different temperatures. **(B)** Growth curve of ZY-1 at different pH. **(C)** Emulsification of ZY-1 under different hydrocarbons. **(D)** Degradation diagram of n-alkanes in crude oil.

The morphology of ZY-1 on LB solid medium at 6% NaCl is shown in Supplementary Figure 1A. By observing the morphology of bacterial colonies on the plates, we can see that the colony appears white and shows a litter transparent in color with a smooth surface. By detecting the optimal growth temperature and pH, it can be seen from Figure 1 that the optimal growth temperature of strain ZY-1 is 30°C (Figure 1A), and the optimal growth pH of strain ZY-1 is 8.5 when under the optimal temperature conditions (Figure 1B). In addition, it could grow at pH 10.5, which revealed that ZY-1 had good alkali resistance, suggesting the potential function when under alkaline conditions. It has been reported that several bacteria are able to generate extracellular compounds to establish their niche and survive (Remonsellez et al., 2018). In the other words, bacteria often use biofilm as a structure to resist adverse conditions. Here, we found that ZY-1 could generate the biofilm under different salt conditions, especially under 5% NaCl (w/v; Supplementary Figure 1B). In the genome of ZY-1, some genes, like *bifA*, *mucR*, *pgaC*, *icaA*, *fliA*, *csrA*, *flgM*, and *tarA*, were found, which belonged to the cellular process were related to the function of biofilm formation. In addition, some genes including *luxS*, *bcsA*, and *wecB* related to metabolism and the formation of biofilm are also found.

To evaluate the emulsifying performance of ZY-1, different petroleum hydrocarbons were selected. From Figure 1C, we can see that the surfactant metabolized by ZY-1 could emulsify C12, C16, benzene, xylene, atoleine, and cyclohexane with good emulsification performance. Moreover, some common carbon sources like glycerol, liquid wax, and sodium acetate, even some hexadecane, octadecane, and dodecane in petroleum hydrocarbons were used and tested. From Supplementary Figure 1C, we can see that ZY-1 not only could use these common carbon sources for metabolic but also can use C12, C28, and C36 to grow, which showed well growth performance when cultured for more than 3 days at the carbon source of C16. Overall, these results showed that ZY-1 could use some n-alkanes for metabolic, which shows potential in the degradation of petroleum hydrocarbon or in the bioremediation of petroleum hydrocarbon pollutants. To further prove that ZY-1 could degrade petroleum hydrocarbons even under a saline environment, crude oil was used to culture ZY-1 for 7 days, and the results of gas chromatography showed that ZY-1 could degrade n-alkanes (degradation rate is about 10%) in petroleum hydrocarbon to a certain extent when under 6% NaCl conditions (Figure 1D). At this point, a conclusion has been drawn that the salt-tolerant strain ZY-1 can not only emulsify petroleum hydrocarbon compounds well but also degrade petroleum hydrocarbon compounds to a certain extent.

**FIGURE 2:**
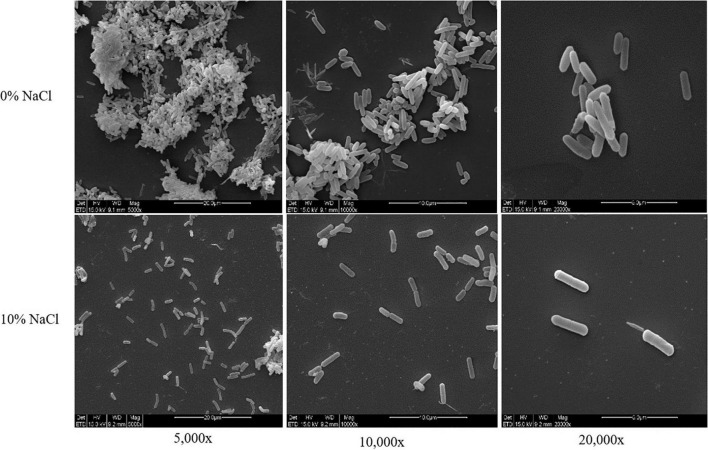
Morphological structure diagram of *T*. *saccharophilus* ZY-1 at 0% and 10% NaCl on SEM at the magnification of 5,000×, 10,000×, and 20,000×.

To explore the morphological structure of ZY-1 under different salinities (0% NaCl, which means normal LB medium; 10% NaCl, which means an additional 10% NaCl was added to the normal LB medium), SEM was employed to observe the morphological feature. In Figure 2, we can see that the morphology of ZY-1 at different magnifications including 5,000×, 10,000×, and 20,000× were all shown and characterized. At the magnification of 20,000×, the bacteria are all rod-shaped at 0% and 10% salinity, ranging in size from 1–2 to 2–3 μm, respectively. It can be seen that the cells become slightly larger with an increase in salinity; in other words, ZY-1 could tolerate different levels of stress, such as salinity by adjusting cell size appropriately. A previous study has shown that the expression of shape-determining genes *mreD* and *mreB* was variably affected at different levels of salinity, which alternatively influenced the diameter of cells (Patel et al., 2018). As salinity directly affects the cell morphology of bacteria, based on the complete genome of ZY-1, some genes related to rod shape-determining protein including *mreD*, *mreC*, *mreB*, and *rodA* are also found and annotated, which may play protective role in bacteria under extreme conditions.

In addition, the phenotypic features of strain ZY-1 were all compared with the other two types of *Terribacillus* species 002-048^T^ and 002-051^T^ (An et al., 2007). On the one hand, from Supplementary Table 1, we can see that except L-Arabinose, other carbohydrates like glucose, sucrose, melibiose, trehalose, and raffinose can be metabolized by these three strains ZY-1, 002-048^T^, and 002-051^T^. It is also worth mentioning that D-galactose could not be metabolized by ZY-1. In addition, other carbohydrates including mannitol, fructose, and mannose were proved to be metabolized by ZY-1. On the other hand, the results of the API-ZYM system showed that ZY-1 has the activity of exogenous enzymes including alkaline phosphatase, esterase (C4), esterase lipase (C8), leucine arylamidase, valine arylamidase, chymotrypsin, acid phosphatase, naphthol-AS-Bi-phosphatase, α-galactosidase, β-galactosidase, β-glucosidase, and naphthol-AS-Bi-phosphatase (Supplementary Table 2).

By comparing the salt tolerance of the three strains, we can see that the maximum tolerance of NaCl for ZY-1, 002-048^T^, and 002-051^T^ were 18, 16, and 19%, respectively, that is, all the three strains belong to moderately salt-tolerant microorganisms, but there still exist some differences in salt tolerance among the three species. Furthermore, strain 002-051^T^ shows better salt tolerance than others. By analyzing the genome of ZY-1, two kinds of prokaryotic-type ABC transporter including glycine betaine/proline (*proW*, *proX*, and *proV*) and osmoprotectant (*opuBB*, *opuBC*, and *opuBA*) were found. As we all know, the organic osmotic protection mechanism is one of the commonly reported mechanisms of salt tolerance (Gutierrez and Devedjian, 1991). When bacteria exist in the extreme salt environment, they could accumulate compatible substances, such as glycine betaine, and proline betaine, which are conducive to the formation of the hydrated outer layer of proteins to maintain normal configuration and function. In addition, an alkanesulfonate transporter (*ssuA*, *ssuB*, and *ssuC*) was found in the genome of ZY-1, the ssu system including the periplasmic alkane sulfonate-binding protein (*ssuA*; Fabiano et al., 2013), the membrane permease protein (*ssuC*), and the nucleotide-binding protein (*ssuB*), which usually mediates the uptake of other aliphatic sulfonates (Ploeg et al., 1996; van Der Ploeg et al., 1999).

### General Genome Features

**FIGURE 3:**
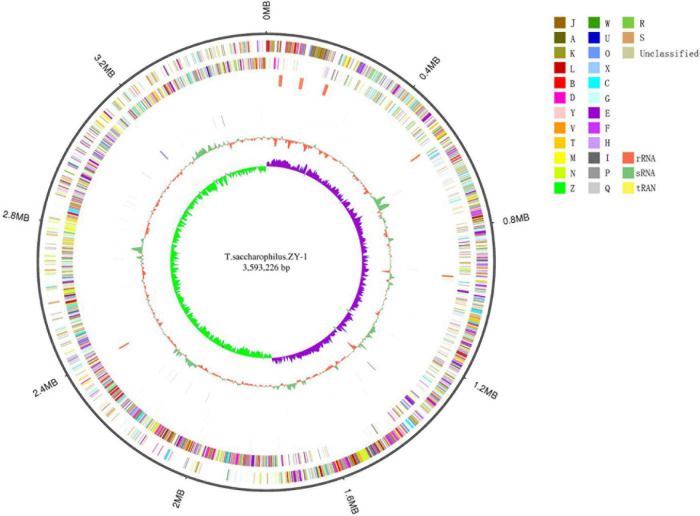
Genome circles of *T. saccharophilus* strains ZY-1. From outer to inner is: genome size; forward strand gene, colored according to cluster of orthologous groups (COG) classification; reverse strand gene, colored according to cluster of orthologous groups (COG) classification; forward strand ncRNA; reverse strand ncRNA; repeat; GC; GC-skew.

In this study, 8,738,130 raw reads covering a total of 3,421,555,660 bp with 230 genome coverage for strain ZY-1 were obtained. A complete chromosome contained a circular molecule of 3,593,226 bp with 42.80% GC content. A total of 3,848 genes were predicted to be coding DNA sequences (CDS), and 3,743 genes were annotated into the database among these genes. Furthermore, 76 tRNA genes, 28 rRNA genes, and 4 sRNA genes were included. The detailed genomic information of ZY-1 and the other five *T. saccharophilus* strains are all listed in Table 1, and the genome circle is shown in Figure 3.

**TABLE 1:** Detailed information of chromosomal genomes from *T. saccharophilus* strain ZY-1 and other five *T. saccharophilus* strains.

Features	ZY-1	7521	7518	7503-2	7517-W	7528
Site of isolation	Produced water	Uncut heroin sample	Uncut heroin sample	Uncut heroin sample	Uncut heroin sample	Uncut heroin sample
Total length (Mb)	3.59323	3.71255	3.7432	3.56601	3.59514	3.55723
GC (%)	42.8	42.9	42.9	42.8	42.8	42.8
Total gene numbers	3,848	3,740	3,779	3,546	3,598	3,536
CDS	3,848	3,741	3,816	3,582	3,634	3,589
tRNA; rRNA	76; 28	59; 6	48; 7	59; 10	55; 8	51; 10
NCBI reference sequence	This work	NZ_NPBP01000006.1	NZ_NPBL00000000.1	NZ_NPBD01000007.1	NZ_NPBK01000001.1	NZ_NPBV01000002.1

In addition, the results of the distribution diagram for COG function annotation are shown in Supplementary Figure 2. In ZY-1, 2,945 (76.53%) genes were annotated. It can be seen that in the four main categories of functions, the gene numbers are more included in metabolism and cellular, some included in information and poorly. In metabolism, there are 328 genes that are related to amino acid transport and metabolism and in cellular, and 226 genes are associated with signal transduction metabolism. From poorly understood information, we identified 280 and 289 genes which are respectively related to transcription and general prediction function. Remarkably, there are still 153 genes whose functions are unknown and 125 genes which are relevant to lipid transport and metabolism.

Studying these functional genes, on the one hand, will help better understand the performance of ZY-1, and on the other hand, it will lay a foundation for the application of genetic engineering or other methods to further modify strains.

### Genome Comparison

To better understand the differences between ZY-1 and other same genus strains, the whole-genome sizes, GC content, and gene contents of these six *T. saccharophilus* strains were all compared. It was clear in Table 1 that except for ZY-1 which was isolated from the reservoir water, the other five strains selected were all isolated from the uncut heroin samples (Kalinowski et al., 2018). In addition, it can be seen that there exist some slight differences among them. In the meantime, the number of total genes increased with the enlargement of genome size (total length). Except strains 7518 and 7521, the GC content of the other four strains is 42.8%. The total gene numbers of ZY-1 are 3,848, which are greater than those in the other five *T. saccharophilus* strains. In addition, the other characteristics including CDs, total gene numbers, tRNA, and rRNA are listed in Table 1.

### Structural Variation Collinearity

In this study, the differences in nucleotide and amino acid levels between ZY-1 and other different strains were compared, respectively, and the results of each level were analyzed and are shown in Supplementary Figures 3, 4. At the nucleotide level, changes in genetic gene loci caused by rearrangement and transposition between the sequenced genome and the reference genome were analyzed. Collinearity analysis at the nucleotide level can reveal insertion, deletion, and other information that can be added to the sequence when compared to the amino acid level (Passarge et al., 1999).

In Supplementary Figure 3, we can see that the collinearity of *T. saccharophilus* ZY-1 was compared with that of the other five strains, respectively. These six maize genomes are very closely related when at the nucleotide level. Furthermore, the genomes of 7517-W and ZY-1 are most similar (Supplementary Figure 3B) as genome-wide collinearity analyses are very valuable for functional prediction. Alignment at the nucleotide level across the genome could help predict the function of coding and non-coding regions. At the level of amino acids, changes in genetic gene loci caused by repeats and transpositions between the sequencing genome and the reference genome can be analyzed (Passarge et al., 1999). From Supplementary Figure 4, we can see that the collinearity of *T. saccharophilus* ZY-1 was compared, respectively, with that of the other five strains at the amino acid level. Compared with Supplementary Figure 3, it can be seen that the collinearity at the amino acid level showed more similarity. As only ZY-1 was the complete genome, there still exist some differences that can be seen in the common genes and specific genes.

Through collinearity analysis, we can obtain the structural variation of inter-species genomes in the process of evolution, such as the change in the position of gene clusters with similar functions in different species. To some extent, collinearity reflects the kinship in the evolutionary process of species. The higher the collinearity is, the closer the kinship will be. The size of colinear fragments is closely related to the time of differentiation among species. Species with short differentiation time have less accumulated variation and retain more traits inherited from their ancestors. On the contrary, species with longer differentiation time have fewer common features due to variation accumulation, but they result in shorter colinear fragments.

### Common Genes and Specific Genes

**FIGURE 4:**
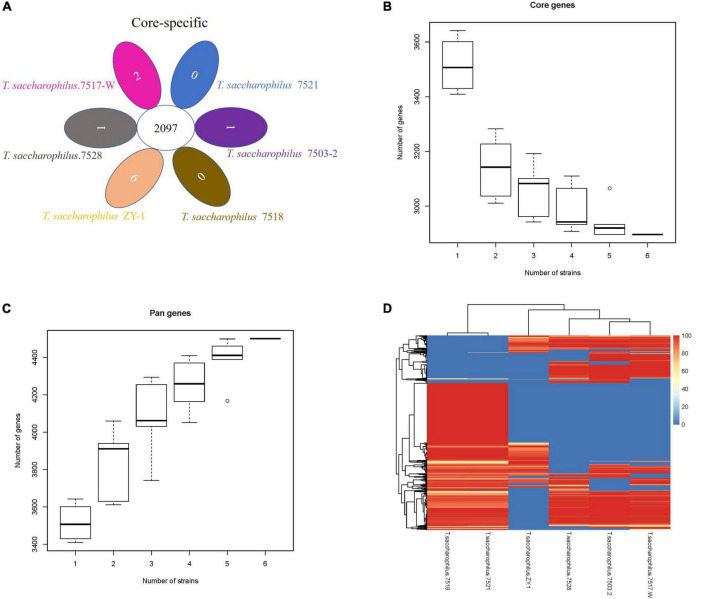
**(A)** Venn diagram of the number of homologous gene families in each strain. **(B)** Core gene dilution curve. **(C)** Pan-gene dilution curve. **(D)** Dispensable gene heat map.

In this study, one scheme was adopted to analyze the common genes and unique genes among strains, and genes at protein levels were selected for processing by default. By analyzing and comparing *T. saccharophilus*.7503-2, *T. saccharophilus*.7517-W, *T. saccharophilus*.7518, *T. saccharophilus*.7521, *T. saccharophilus*.7528, and *T. saccharophilus* ZY-1, we can see that the common genes and specific genes are easier to identify. Moreover, the numbers of total genes, filtered genes, and final genes are shown in Supplementary Table 3. In addition, the common and special genes among strains of *T. saccharophilus* ZY-1, the gene set of each strain, and the statistical results are shown in Figure 4A.

The protein gene sets of all strains were analyzed by CD-HIT v4.6.6 cluster analysis, and the final gene set of the cluster was regarded as a pan-gene set. The sequences of each sample in the cluster were extracted as a core gene set, and the specific gene set in the product is a specific gene set. The pan-gene set is composed of core gene sets, and the specific gene set is a dispensable gene set. From the perspective of evolution, core genes may be the genes that perform key functions, which tend to be some conserved genes in a species. On the contrary, non-core genes promote the diversity of species and enable them to adapt to various environmental conditions. The dilution curve for core genes and pan-genes are shown in Figures 4B,C, respectively. Both the horizontal axis represents the number of samples selected for each statistic, and the vertical axis represents the number of genes (from bottom to top, it is composed of five numerical points: minimum (min), lower quartile (Q1), median, upper quartile (Q3), and maximum (Max), where the lower quartile, median, and upper quartile form a box with compartments).

From Figure 4B, we can see that with the increase in the strain number, the number of core genes gradually decreased. When the number of strains is six, the number of core genes is 2,896 (core gene size: 875,495 bp). On the contrary, from Figure 4C, we can see that the number of pan-genes increased gradually with the increase of strain numbers, the number of pan-genes is 4,501 (pan-gene size: 1,275,981 bp) when the number of strains is six. Previous studies have shown that the size of the pan-genome gradually increased with the number of comparative strains increased, while the size of the core genome stabilized (Mikalsen et al., 2015). Core genes are usually associated with basic cellular functions that control basic metabolic processes in living organisms, while non-core genes are associated with environmental and defense responses, receptor and antioxidant activity, gene regulation, and signal transduction. Through functional annotation, we can understand the function of the core, variable, and endemic gene families, which provide a basis for finding the pathways related to the formation of specific functions and traits in species. As the number of our comparative strains is six, there will be a clear trend if they have enough strains. In addition, according to the distribution of dispensable genes in different samples, heat maps were drawn to show the clustering among samples (Figure 4D). From the clustering tree, we can see that there exist some differences among the six strains. In addition, many genes in ZY-1 showed lower coverage, which exhibited more diversity. The study of common genes and unique genes, on the one hand, plays an important role in exploring the functional differences and similarities among different genomes. On the other hand, it also provides a molecular basis for the study of similarities and differences of species phenotypes.

### Gene Family

A group of genes derived from the same ancestor and composed of two or more copies of a gene produced by gene replication. They are often similar in either structure or function. Gene families encoding similar protein products can be used to measure the history of biological evolution and the differentiation of gene functions. At the same time, it can be used to predict the function of unknown proteins for the similarity of gene family member functions. The members of gene families usually have a similar function, and they may be from the same gene replication (collateral), which is also likely to come from the same gene evolution (orthologous) when the source of the same genes is closely packed in the chromosomes of an area. It will form the gene cluster, but family members are usually scattered in the genome. Furthermore, as new gene families come from the evolutionary process of organisms, by combining with phylogenetic analysis, gene families can be used to measure the evolutionary history of organisms and find the differentiation of gene functions, etc. It can also be used to predict the function of unknown proteins for the similarity in function of gene family members (Demuth et al., 2006). In this study, the gene family of the six strains was analyzed and compared, and the concrete results are shown in Table 2.

**TABLE 2:** Statistics of gene family for the six strains.

Sample name	Gene number	Clustered gene	Unclustered gene	Family number	Unique family
*T. saccharophilus*. ZY-1	3,848	3,523	325	2,263	9
*T. saccharophilus*.7503-2	3,546	3,480	66	2,356	1
*T. saccharophilus*.7517-W	3,598	3,530	68	2,374	2
*T. saccharophilus*.7518	3,779	3,738	41	2,512	0
*T. saccharophilus*.7521	3,740	3,730	10	2,505	0
*T. saccharophilus*.7528	3,536	3,460	76	2,353	1

*Gene number, gene number of each strain; Clustered genes, the number of genes that cluster into gene families; Unclustered genes, genes that are not clustered into any family; Family num, number of gene family of strain; and Unique gene family, the number of unique gene family to strains.*

From Table 2, we can see that their gene numbers are all between 3,500 and 3,900, and the clustered genes are between 3,400 and 3,800. In addition, some unclustered genes are less than 80, except ZY-1 (325); moreover, the family numbers are between 2,200 and 2,600, and the unique family is 9 (ZY-1), 1 (7503-2), 2 (7517-W), 0 (7518 and 7521), and 1 (7528), respectively. Compared with their gene families, we can see that there exist some differences among the six strains. Further studies on genes of these different families are expected to provide a basis for revealing the causes of phenotypic differences among the six strains. It is worth noting that the study of these nine unique families in ZY-1 is expected to further reveal the biological characteristics of ZY-1.

**FIGURE 5:**
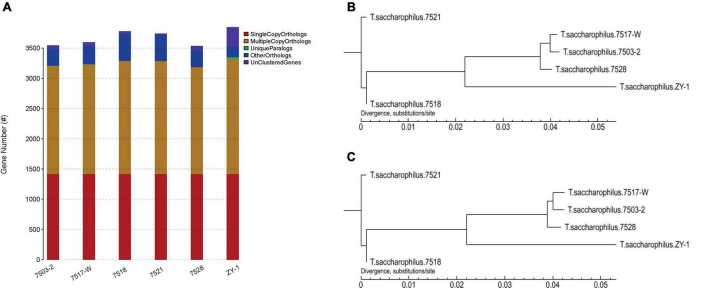
**(A)** Comparison of gene family composition of six strains. **(B)** Phylogenetic trees based on CorePan1. **(C)** Phylogenetic trees based on GeneFamily1.

Furthermore, the gene family compositions are compared in Figure 5A; the single copy orthologs, multiple copy orthologs, unique paralogs, other orthologs, and unclustered genes are all listed in Table 2. We can see that there is no increased difference in single copy orthologs, which existed in all these six strains. It is worth noting that there obviously exist some unique paralogs, and compared with other strains, there are more unclustered genes in ZY-1, which are usually associated with some biological phenomena unique to the species. As paralogs are proteins derived from gene replication in a given species that may evolve new functions associated with the original, these unique paralogs will help discover some new genes, which showed good potential to evolve other new functions.

### Evolution of Species

Cladistic diagrams drawn between different species based on phenotypic or genotypic similarities and differences can be used to describe the evolutionary relationships among different species. In systematics, it may play an important role in the research of species evolution. In a tree, each node usually represents the most recent common ancestor of each branch, and the length of the line segment between the nodes corresponds to the evolutionary distance (such as the estimated evolutionary time). Trees are usually divided into root and rootless trees.

Here, the software of TreeBeST v1.9.2 was used to build phylogenetic trees. This study is based on CorePan1 and GeneFamily1 analysis methods, and the two phylogenetic trees are shown in Figure 5, which were constructed by using the two methods, respectively. We can notice that based on CorePan1 (Figure 5B) or GeneFamily1 (Figure 5C), the phylogenetic trees are almost similar. The strains including 7517-W and 7503-2 are most closely related in evolution. In addition, ZY-1 showed more closest evolutionary distance among 7517-W, 7503-2, and 7528, which indicated that the four strains were relatively closest in terms of evolutionary relationship. The long evolutionary distance between ZY-1 and other strains indicated that ZY-1 has some differences with other strains in the evolutionary process, and the long evolutionary distance also suggested that ZY-1 might have more different functions than other strains from the same genus.

## Conclusion

In this study, a salt-tolerant bacterium named *T. saccharophilus* ZY-1 was isolated from reservoir water. For ZY-1, the physicochemical properties and morphological structure at different salinity conditions were studied, and the complete genome was sequenced and compared. As previous studies of *T. saccharophilus* have paid less attention to the study of their complete genome, this article first reported the complete genome of *T. saccharophilus* species with salt tolerance and petroleum hydrocarbon degradation and emulsifying properties, which not only showed the characteristics of high salt-tolerance but also have the ability to degrade crude oil and even emulsify some petroleum hydrocarbons. ZY-1 was confirmed to belong to *T. saccharophilus* from Bacillus with high salt tolerance, and its genome sequences of ZY-1 were further compared with those of the other five strains. Due to the high salinity of the reservoir environment.

The oil leakage caused by oil reservoir production also produces a series of environmental pollution problems, such as soil pollution, water pollution, air pollution, and marine ecological pollution. Therefore, the excavation and application of high salt-tolerant bacteria can be used not only for oil recovery but also for bioremediation of high-salinity polluted environments. Compared with other methods, biological methods seem to be simple and green and follow the environmental protection concept of taking from nature and using from nature, which is more suitable for future environmental governance. In addition, when sucrose was used as the carbon source, the emulsifying performance of the surfactant was tested through the fermentation and the emulsifying properties of different petroleum hydrocarbons, which will provide a research basis for further digging and exploring emulsifying mechanisms. On the one hand, it was found that the surfactant produced by ZY-1 had a better emulsifying performance than petroleum hydrocarbons with a wide range of emulsifying substrates. On the other hand, as ZY-1 was isolated from reservoir produced water, the results of degradation performance of strain to petroleum hydrocarbon will provide a good reference value for researchers in the field of petroleum microbiology. In addition, collinearity between the other five strains and ZY-1 was analyzed by comparing the gene families, core genes, and pan-genes, which are expected to further reveal the evolutionary relationship between ZY-1 and other strains.

Through the analysis and comparison of genomic data, it is expected to provide guidance and direction for further revealing the properties like salt tolerance, degradation, and emulsification of petroleum hydrocarbon for ZY-1. Overall, the study of highly salt-tolerant strains ZY-1 is expected to further realize the bioremediation of petroleum pollutants and other environments. By comparing the genomes of different strains from the same genus, the evolutionary relationship between strains can be further analyzed, and some unique and common genes of strains can also be excavated and found. In other words, these findings will provide a reference for further research on the function of *T. saccharophilus* and provide support for further broadening the function of *T. saccharophilus* in the environment.

## Data Availability Statement

The data presented in this study are deposited in the NCBI repository, access number: CP096209.

## Author Contributions

ZS and SW performed the data processing and handling. ZS wrote the manuscript. ML, SY, YC, and YY were modified. TM and GL conducted the project planning. All authors contributed to the article and approved the submitted version.

## Conflict of Interest

The authors declare that the research was conducted in the absence of any commercial or financial relationships that could be construed as a potential conflict of interest.

## Publisher’s Note

All claims expressed in this article are solely those of the authors and do not necessarily represent those of their affiliated organizations, or those of the publisher, the editors and the reviewers. Any product that may be evaluated in this article, or claim that may be made by its manufacturer, is not guaranteed or endorsed by the publisher.

## Footnotes

1
https://github.com/marbl/canu/releases

2
https://www.broadinstitute.org/gatk/

3
http://rfam.sanger.ac.uk/

4
https://tygs.dsmz.de/

5
http://mummer.sourceforge.net/
