# Supplementary material for: Complete Genome Sequences of One Salt-Tolerant and Petroleum Hydrocarbon-Emulsifying Terribacillus saccharophilus Strain ZY-1
Source: Front Microbiol. 2022 Jul 28;13:932269. doi: 10.3389/fmicb.2022.932269 (PMC9366552; doi:10.3389/fmicb.2022.932269)
Supplement: Supplementary file 1 [file Data_Sheet_1.docx]

***Supplementary Material***

1.
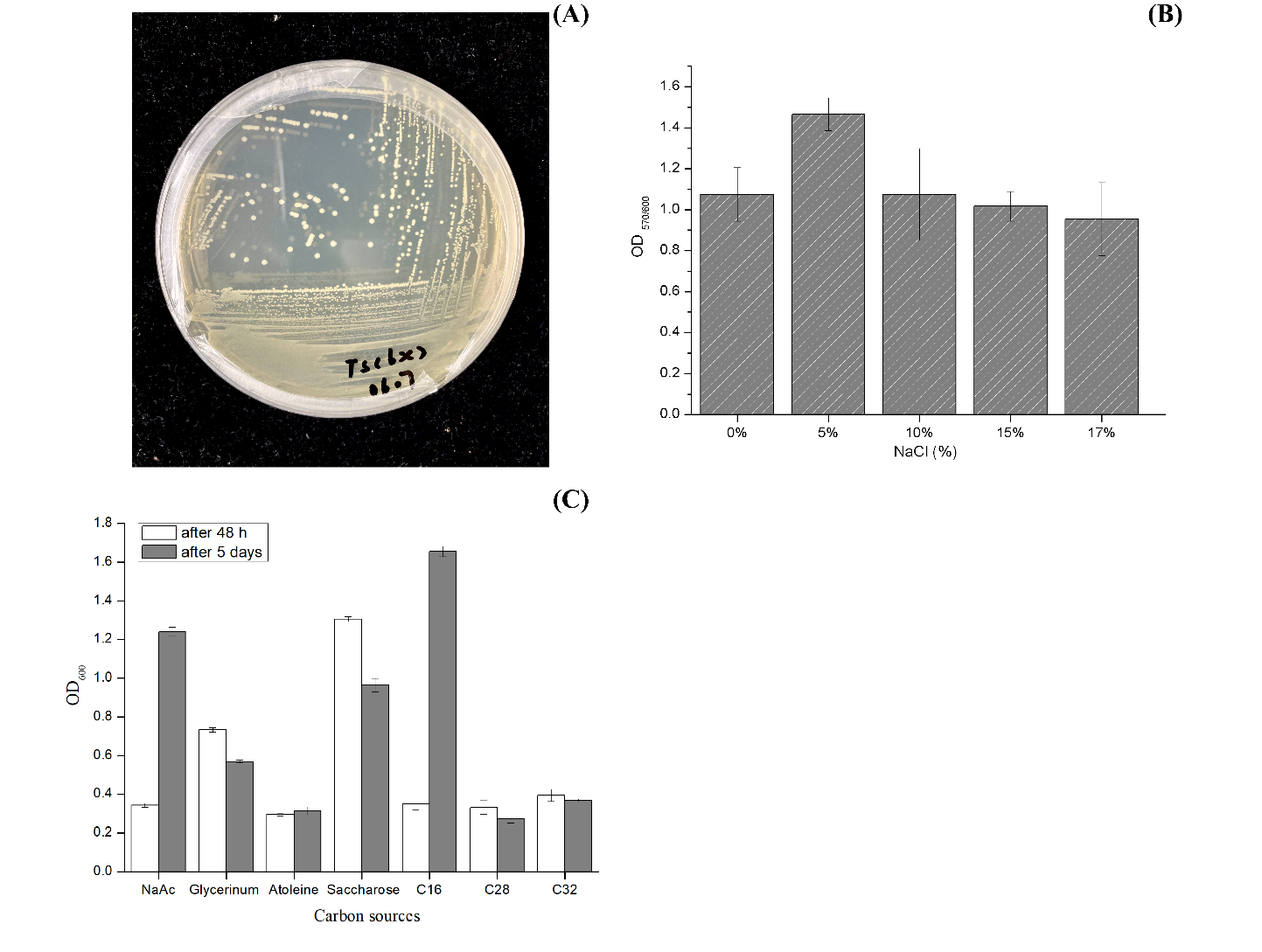
Supplementary Figures

**Supplementary Figure 1. (A)** Morphology of *T. saccharophilus* ZY-1 on solid LB plate at 6% NaCl **(B)** Biofilms formation under different salt concentrations **(C)** Utilization of different carbon sources

**Supplementary**
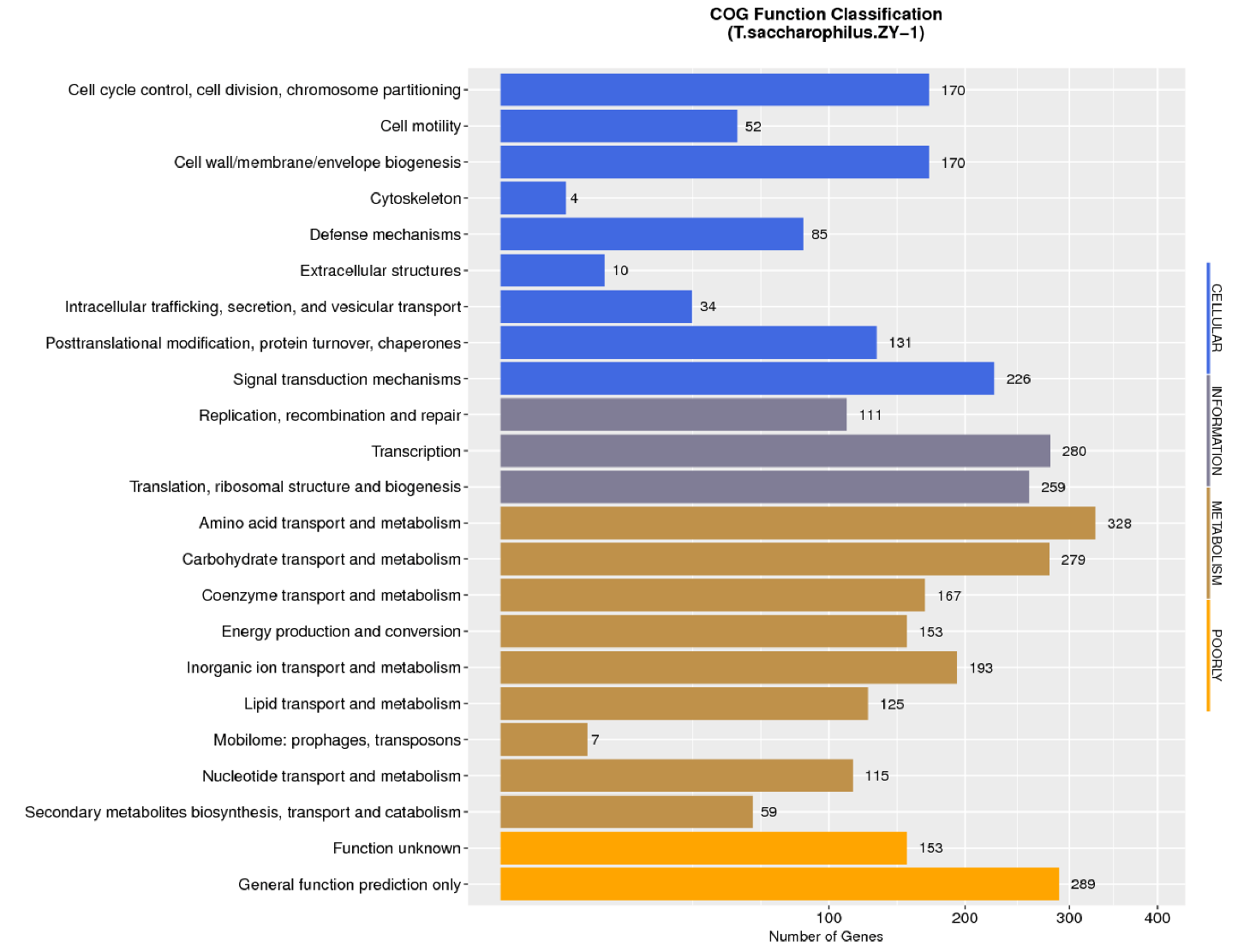
**Figure 2.** COG pathway classification

**
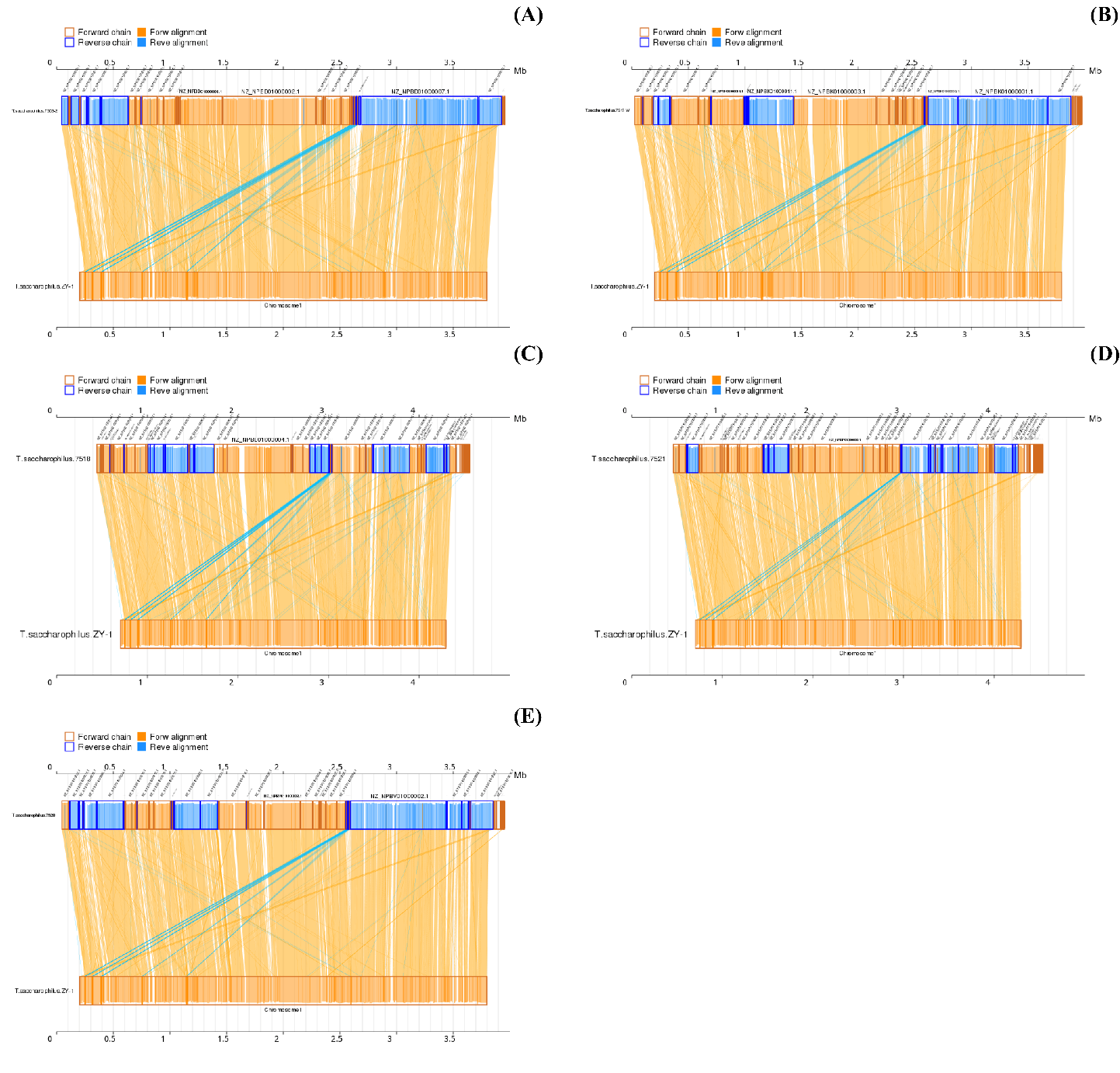
**

**Supplementary** **Figure 3.** Compared the differences between strains at nucleotide level. (**A**) 7503-2 and ZY-1 (**B**) 7517-W and ZY-1 (**C**) 7518 and ZY-1 (**D**) 7521 and ZY-1 (**E**) 7528 and ZY-1 (The lower sequence represents the measured genome, the upper sequence represents the reference sequence, and the yellow box represents the positive strand of the genome, and the blue box represents the antistrand of the genome. The yellow area in the box indicates that the amino acid sequence in this region of the genome is in the positive chain, and the blue area indicates that the amino acid sequence in this region of the genome is in the negative chain diagram. The yellow line in the middle of the two sequences indicates the forward alignment, and the blue line indicates the reverse complementary alignment.)

**(E)**

**(B)**

**(E)**

**(D)**

**Supplementary**
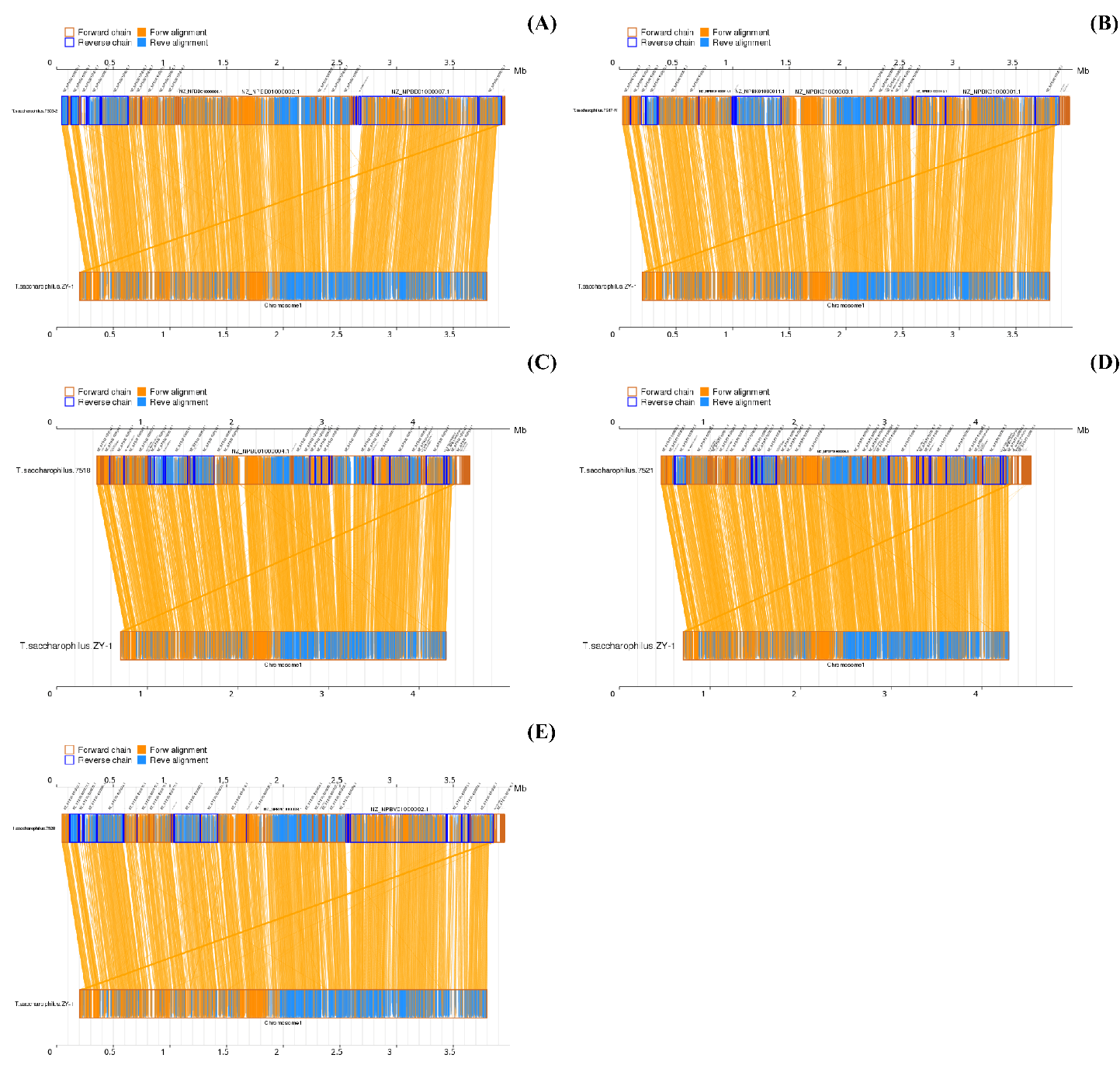
**Figure 4.** Compared the differences between strains at amino acid level. (**A**) 7503-2 and ZY-1 (**B**) 7517-W and ZY-1 (**C**) 7518 and ZY-1 (**D**) 7521 and ZY-1 (**E**) 7528 and ZY-1 (The lower sequence represents the measured genome, the upper sequence represents the reference sequence, and the yellow box represents the positive strand of the genome, and the blue box represents the antistrand of the genome. The yellow area in the box indicates that the amino acid sequence in this region of the genome is in the positive chain, and the blue area indicates that the amino acid sequence in this region of the genome is in the negative chain diagram. The yellow line in the middle of the two sequences indicates the forward alignment, and the blue line indicates the reverse complementary alignment.)

2. Supplementary Tables

**Supplementary Table 1** Phenotypic features for different carbohydrates of ZY-1 and other two types of *Terribacillus* species.

| **Characteristics** | **ZY-1** | **002-048^T^** | **002-051^T^** |
| --- | --- | --- | --- |
| D-glucose | + | + | + |
| D-sucrose | + | + | + |
| L-arabinose | - | - | - |
| D-galactose | - | + | + |
| D-melibiose | + | + | + |
| D-trehalose | + | + | + |
| D-raffinose | + | + | + |
| Mannitol | + | / | / |
| D-Fructose | + | / | / |
| D-Mannose | + | / | / |
| N-acetylglucosamine | + | / | / |
| Salicin | + | / | / |
| D-Cellobiose | + | / | / |
| D-gentiobiose | + | / | / |
| D-tager sugar | + | / | / |
| Maximum tolerable of NaCl (%) | 18 | 16 | 19 |
| Reference | This work | (An et al., 2007) | (An et al., 2007) |

+, Positive; -, negative; /, unclear

**Supplementary Table 2** The physiological and biochemical characteristics for the activity of exogenous enzyme of ZY-1.

| **Characteristics** | **ZY-1** |
| --- | --- |
| Alkaline phosphatase | + |
| Esterase (C4) | + |
| Esterase lipase (C8) | + |
| Lipase (C14) | - |
| Leucine arylamidase | + |
| Valine arylamidase | + |
| Cysteine arylamidase | - |
| Trypsin | - |
| Chymotrypsin | + |
| Acid phosphatase | + |
| Napthol-AS-Bi-phosphatase | + |
| α-galactosidase | + |
| β-galactosidase | + |
| β-glucuronidase | - |
| α-glucosidase | - |
| β-glucosidase | + |
| N-acetyl-β-glucosaminidase | - |
| α-mannosidase | - |
| β-fucosidase | - |

+, Positive; -, negative;

**Supplementary Table 3** Statistical table of genes of each strain

| Sample Name | Total Gene Num | Filtered Gene Num | Final Gene Num |
| --- | --- | --- | --- |
| *T. saccharophilus*. ZY-1 | 3,848 | 0 | 3,848 |
| *T. saccharophilus*.7503-2 | 3,546 | 2 | 3,544 |
| *T. saccharophilus*.7517-W | 3,598 | 6 | 3,592 |
| *T. saccharophilus*.7518 | 3,779 | 6 | 3,773 |
| *T. saccharophilus*.7521 | 3,740 | 8 | 3,732 |
| *T. saccharophilus*.7528 | 3,536 | 8 | 3,528 |

Reference

An, S.Y., Asahara, M., Goto, K., Kasai, H., and Yokota, A. (2007). Terribacillus saccharophilus gen. nov., sp. nov. and Terribacillus halophilus sp. nov., spore-forming bacteria isolated from field soil in Japan. *International Journal of Systematic Evolutionary Microbiology* 57(1)**,** 51-55.
